# Not just a research method: If used with caution, can job-exposure matrices be a useful tool in the practice of occupational medicine and public health?

**DOI:** 10.5271/sjweh.3900

**Published:** 2020-09-01

**Authors:** Marc Fadel, Bradley A Evanoff, Johan H Andersen, Angelo d’Errico, Ann Marie Dale, Annette Leclerc, Alexis Descatha

**Affiliations:** 1 UNIV Angers, CHU Angers, Univ Rennes, Inserm, EHESP, Irset (Institut de recherche en santé, environnement et travail) - UMR_S1085, CAPTV CDC, Angers, France; 2 Division of General Medical Sciences, Washington University School of Medicine, St. Louis, Missouri, USA; 3 Occupational Medicine, University Research Clinic, Herning, Denmark; 4 Unit of Epidemiology, Regional Health Service ASL TO3, ­Grugliasco (Turin), Italy; 5 Inserm, UMS 011 Population-based Epidemiologic Cohorts Unit, Villejuif, France

The recent editorial by Dr Susan Peters “Although a valuable method in occupational epidemiology, job-exposure matrices are no magic fix” ably describes the strengths and limitations of job-exposure matrix (JEM) approaches in occupational epidemiology research (1). In addition to their use in research, we would like to add that JEM may also be of use in compensation and surveillance efforts in occupational health.

JEM could assist the compensation process by supporting the assessment of relevant exposures related to specific health conditions (2). The potential usefulness of a JEM as a decision tool for compensation of work-related musculoskeletal disorders has been examined (3). Because occupational diseases are often under-recognized, another practical application is using a JEM to screen for occupational exposures as part of health surveillance. Use of JEM to screen for asbestos and wood dust exposure in the clinical setting has shown promising results (4–6). By summarizing multiple exposures at a job level (7), JEM may also assist policy-makers in setting priorities for hazards and controls at work, as well as occupational practitioners to target prevention efforts and direct the conduct of more precise exposure measures to particular jobs.

Sharing JEM across different countries may be useful in providing estimates of exposures across larger populations to calculate global burden of disease related to occupational exposure. The JEMINI (JEM InterNatIonal) initiative was launched to explore the possibility of developing international JEM that could be used across countries (8). Beginning with physical (biomechanical) exposures, this open group has started homogenizing job coding systems and comparing some available JEM. Estimating differences in the level of exposure between countries will require much more work, without guaranteed success.

As Peters mentioned, many limitations exist in the use of JEM. Users of JEM must consider the source of exposure data – expert assessments, data collected from individual workers, or environmental sampling. The coding of occupations is time consuming and can introduce error (9), and more testing of and comparison with automated job coding systems is needed (10). JEM reflect an “average” level of exposure within a job at the expense of individual variation. At population level, JEM can offer a useful estimate of exposures. If used at an individual level in a clinical or compensation setting, JEM cannot replace the professionals involved in exposure assessment but may help them focus their action more effectively on complex situations that require their expertise.

In conclusion, these JEM developed for research might also be used as a public health tool, provided that their limitations are properly taken into account.
